# Identification of Small Molecules Affecting the Secretion of Therapeutic Antibodies with the Retention Using Selective Hook (RUSH) System

**DOI:** 10.3390/cells12121642

**Published:** 2023-06-16

**Authors:** Mathilde Coulet, Sylvie Lachkar, Marion Leduc, Marc Trombe, Zelia Gouveia, Franck Perez, Oliver Kepp, Guido Kroemer, Stéphane Basmaciogullari

**Affiliations:** 1 Sanofi R&D, 94400 Vitry-sur-Seine, France; 2 Metabolomics and Cell Biology Platforms, Gustave Roussy Cancer Center, 94800 Villejuif, France; 3 Centre de Recherche des Cordeliers, Equipe Labellisée par la Ligue Contre le Cancer, Université de Paris Cité, Sorbonne Université, Inserm U1138, Institut Universitaire de France, 75006 Paris, France; 4 Institut Curie, PSL Research University, Sorbonne Université, CNRS, UMR 144, 26 rue d’Ulm, 75005 Paris, France; 5 Department of Biology, Institut du Cancer Paris CARPEM, Hôpital Européen Georges Pompidou, AP-HP, 75015 Paris, France

**Keywords:** RUSH assay, antibody, high-throughput screening, protein secretion

## Abstract

Unlocking cell secretion capacity is of paramount interest for the pharmaceutical industry focused on biologics. Here, we leveraged retention using a selective hook (RUSH) system for the identification of human osteosarcoma U2OS cell secretion modulators, through automated, high-throughput screening of small compound libraries. We created a U2OS cell line which co-expresses a variant of streptavidin addressed to the lumen-facing membrane of the endoplasmic reticulum (ER) and a recombinant anti-PD-L1 antibody. The heavy chain of the antibody was modified at its C-terminus, to which a furin cleavage site, a green fluorescent protein (GFP), and a streptavidin binding peptide (SBP) were added. We show that the U2OS cell line stably expresses the streptavidin hook and the recombinant antibody bait, which is retained in the ER through the streptavidin–SBP interaction. We further document that the addition of biotin to the culture medium triggers the antibody release from the ER, its trafficking through the Golgi where the GFP-SBP moiety is clipped off, and eventually its release in the extra cellular space, with specific antigen-binding properties. The use of this clone in screening campaigns led to the identification of lycorine as a secretion enhancer, and nigericin and tyrphostin AG-879 as secretion inhibitors. Altogether, our data support the utility of this approach for the identification of agents that could be used to improve recombinant production yields and also for a better understanding of the regulatory mechanism at work in the conventional secretion pathway.

## 1. Introduction

Secretion is a fundamental cell-biology process that enables the release of molecules in the extracellular environment. This capacity is necessary to fulfill a multitude of needs, and the released molecules can play roles in growth, chemotactism, homeostasis, immunity, and cell–cell signaling, to mention a few [1]. Eukaryotic cells have a functional network of interconnected organelles, enabling the synthesis and the controlled transport of a variety of proteins along multiple routes. In particular, the conventional secretory pathway allows for the exocytosis of proteins synthetized in the endoplasmic reticulum (ER) to the extracellular environment. Upon completion of mRNA translation and protein translocation to the lumen of the ER, the newly formed proteins are sorted in specialized membrane structures that bud coat protein complex II (COPII)-coated vesicles. These vesicles are transported along the microtubule network towards the Golgi apparatus, where fusion with the *cis*-Golgi takes place, involving the interaction of soluble N-ethylmaleimide-sensitive-factor attachment receptors (SNAREs) present on both the vesicles and *cis*-Golgi membranes. Proteins undergo additional post-translational modifications as they reach the *trans*-Golgi face from which the clathrin-coated vesicles bud. These vesicles transport their payload to the plasma membrane, where SNARE-mediated fusion occurs, and the proteins are eventually released into the extracellular space [2]. In addition to the structural components that make the trafficking of vesicles from the ER to the plasma membrane possible, many regulatory mechanisms act as quality control during this process, such as the unfolded protein response (UPR) within the ER, where misfolded proteins are diverted from the secretory pathway, in order to ensure the secretion of functional proteins. Defects in protein folding and secretion can lead to various pathologies, such as cancer, and neurodegenerative, metabolic, and inflammatory diseases, highlighting the importance of better understanding the underlying cellular mechanisms, which would pave the way for the identification of novel therapeutic molecules [3,4,5,6].

An understanding of the secretion mechanisms is also of paramount interest for the pharmaceutical industry, which has started a transition from the all-chemical era to biologics. Indeed, the proportion of drugs made from living organisms has greatly increased in the past decades, today representing one quarter of all approved therapies; half of them being for therapeutic antibodies [7]. Such antibodies are produced after transfection of suitable cells with specific heavy and light chain-encoding vectors, cell culture upscaling, and antibody purification from the cell culture supernatant [7]. Several directions have been investigated with the final goal of optimizing titers of recombinant proteins, and these include cell host selection, vector design, and medium formulation, together with upstream and downstream process optimization. Small-scale experiments are thus conducted in development labs, before intermediate and final upscaling of the production process in bioreactors for clinical or commercial manufacturing [8]. Besides these canonical optimization efforts, little has been reported in the engineering literature regarding the secretion mechanism itself, from the protein assembly in the ER down to its secretion in the extracellular environment [9]. The identification of modulators of protein secretion is an efficient strategy for enriching the available knowledge on the key elements of the secretory pathway. Among the inhibitors reported in the literature, several small molecules have enabled highlighting the role of the cytoskeleton in protein secretion. Brefeldin A is known for its capacity to impact the anterograde transport of vesicles from the ER to the Golgi, while nocodazole causes depolymerization of microtubules, and cytochalasin B destabilizes the actin network [10,11]. In addition, monensin was shown to block the transport of the protein within the Golgi apparatus [12]. All of these compounds significantly decrease protein secretion and have made it possible to study the key molecular mechanisms of the conventional secretory pathway. Other approaches have also allowed the identification of practices or compounds capable of increasing protein secretion. For example, cell cycle arrest has been correlated with enhanced protein production. Although the exact mechanisms remain to be understood, this strategy is used in the industry to increase production yields. Cell cycle arrest can be achieved through genetic engineering, such as in cell cycle regulatory gene over-expression; through the induction of the hypothermic response; or through the use of small molecules, such as sodium butyrate [13,14]. The importance of chaperone proteins for the correct folding of nascent proteins and secretion has also been widely documented, and their over-expression obtained through genetic manipulation resulted in enhanced protein secretion in several studies [2,15,16]. For example, targeting the UPR via the upregulation of genes involved in this pathway or using ER stress-inducing small molecules resulted in increased cell-specific productivity, confirming the importance of this response [17]. Finally, the conventional secretory pathway was successfully activated through strategies involving the proteins required for the correct addressing of vesicles, such as SNAREs [18], ceramide transferase protein (CERT) [19], and Munc18b [2], or proteins participating in the dynamics of the cytoskeleton, including cofilin [20], actin alpha cardiac muscle 1 (ACTC1), and guanosine triphosphate GTPase-activating protein (TAGAP) [21,22].

In line with these studies, we aimed to identify new modulators of protein secretion by leveraging retention using a selective hook (RUSH) system in a medium-throughput drug library screening approach. The RUSH system was first described by Boncompain et al. [23] and is based on the retention of the protein of interest in a specific subcellular location, until its triggered release in the secretory pathway. This model is particularly useful in a kinetics-based screening approach, where events need to be synchronized. In this work, the RUSH system was adapted to study the secretion of a modified anti-PDL1 antibody and the screening of small compound libraries. A GFP moiety was added to the C-terminal portion of the antibody heavy chain, to allow secretion monitoring with video microscopy. In addition, a furin-cleavable sequence was inserted between the Fc domain of the antibody and the GFP moiety. This ensured the processing of the fusion protein by Golgi-resident furins [24] and the release of a native antibody in the extracellular space that was directly usable in functional assays. Our screen identified inhibitors and activators of antibody secretion, which were further validated in an antigen binding assay.

## 2. Materials and Methods

### 2.1. Cell Culture, Chemicals, and Antibodies

Human osteosarcoma U2OS cells obtained from the Kroemer lab were maintained at 37 °C in a humidified incubator with 5% CO_2_ in Dulbecco’s modified Eagle medium (DMEM) supplemented with 10% fetal bovine serum (FBS), 1% penicillin-streptomycin, and 1% HEPES (Gibco, Carlsbad, CA, USA). CHO-K1 (CCL-61) cells were obtained from the ATCC and cultured at 37 °C in a humidified incubator with 5% CO_2_ in F-12 Ham’s nutrient mixture (Sigma, Saint-Louis, MO, USA) supplemented with 10% FBS and 2 mM L-Glutamine (Gibco). PD-L1-expressing CHO cells (Sanofi property) were cultured in similar conditions in medium supplemented with 7.5 µg/mL puromycin (Gibco). Prestwick, the BML-2840 ICCB Known Bioactives, and the Autophagy Compounds libraries were purchased from Prestwick Chemicals (Illkirch, France), Enzo Life Sciences (Farmingdale, NY, USA), and TargetMol (Boston, MA, USA), respectively. Anti-streptavidin (Santa Cruz Biotechnology, Dallas, TX, USA), anti-calreticulin (Abcam, Cambridge, UK), anti-SBP (Santa Cruz Biotechnology), anti- human heavy and light IgG chains (Invitrogen), and the anti-GFP (Cell Signaling, Danvers, MA, USA) antibodies were used at 1:500, 1:200, 1:500, 1:5000, and 1:1000 dilutions, respectively, in Western blotting and immunofluorescence experiments. The reference anti-human PD-L1 antibody used in FACS experiments was produced at Sanofi labs from cells transfected with plasmid constructs based on the published Atezolizumab heavy and light chain sequences available on the IMGT website (imgt.org, accessed on 12 May 2023) [25]. The secondary Alexa Fluor 568- and 488-conjugated anti-mouse and rabbit IgG antibodies (Life Technologies, Carlsbad, CA, USA) were used at 1:1000 and 1:500 dilutions, respectively, in immunofluorescence and FACS experiments. The secondary HRP-conjugated goat anti-mouse and rabbit IgG (Southern Biotech, Birmingham, AL, USA) antibodies were used at a 1:5000 dilution in the Western blotting experiments. The anti-human kappa light chain antibody (Thermo Fisher Scientific, Waltham, MA, USA) was used at a 1:4000 dilution in ELISA.

### 2.2. Constructs Used for the RUSH Antibody Secretion System

The streptavidin-KDEL plasmid was described in Zhao et al. [26]. The amino acid sequences of the heavy and light chains of the anti-PD-L1 Atezolizumab antibody available on IMGT were used to generate nucleic acid sequences adapted to expression in human cells. Sequences encoding a furin-cleavable site (AVSKERSKRSP), a linker (G_4_S)_2_, enhanced green fluorescent protein (eGPF), and a streptavidin binding protein (SBP) were added in that order to the 3′ end of the anti-PD-L1 heavy chain coding sequence. Anti-PD-L1 heavy and light chain constructs were manufactured by GeneArt (Ratisbonne, Germany) and provided in a shuttle vector. The heavy and light chain sequences were then cloned into the multiple cloning sites 2 and 1, respectively, of the pVITRO1 plasmid (Invivogen, San Diego, CA, USA) using a NEBuilder assembly kit (New England Biolabs, Ipswich, MA, USA). The integrity of the construct, hereafter referred to as anti-PD-L1 IgG RUSH plasmid, was confirmed by sequencing (Eurofins, Luxemburg).

### 2.3. Establishment of the RUSH-Antibody Cell Line

U2OS cells were transfected with the streptavidin-KDEL plasmid using Lipofectamine 2000 (Thermo Fisher Scientific) and selected in medium supplemented with G418 (400 µg/mL, Gibco-Invitrogen) for 2 weeks. Limiting dilution in 96-well plates was used for single cell isolation from the selected pool. Clones were amplified and selected based on streptavidin immunofluorescence. Clone #BD6 showing the highest streptavidin expression was further expanded in medium supplemented with G418 (200 µg/mL) and transfected with the anti-PD-L1 IgG RUSH plasmid using Lipofectamine 2000 and selected in medium supplemented with G418 (200 µg/mL) and hygromycin (100 µg/mL, Santa Cruz Biotechnology) for 2 weeks. Limiting dilution in 96-well plates was used for single cell isolation from clone #BD6-derived selected pool. Clones were amplified and selected based on GFP fluorescence, and clone #7 was selected for further characterization and screening campaigns.

### 2.4. Immunofluorescence

U2OS cells were seeded in black 96-well imaging plates (Greiner bio one, Kremsmünster, Austria). After 24 h, the medium was removed, cells were rinsed with 37 °C pre-heated PBS and fixed in PBS supplemented with 4% paraformaldehyde (PFA, Sigma) and 1 µg/mL Hoechst (Life Technologies, Carlsbad, CA, USA) for 20 min under agitation at room temperature (RT). Cells were rinsed with PBS before addition of a 5% FBS/0.3% Triton X-100/PBS permeabilizing solution for 30 min under agitation at RT. Cells were rinsed with PBS and incubated overnight under agitation at 4 °C with the primary antibody in 1% BSA/PBS. Cells were washed with PBS and incubated for 45 min under agitation at RT with the secondary antibody in 1% BSA/PBS. Finally, cells were washed and processed for image acquisition using a IXM XL BioImager (Molecular Devices, Sunnyvale, CA, USA).

### 2.5. Antibody Expression and Secretion Characterization by Western Blot

U2OS clone #7 cells were seeded in T-175 flasks and kept for 24 h at 37 °C in a humidified incubator with 5% CO_2_. The next day, the supernatant was removed; cells were rinsed with 37 °C pre-heated PBS; and new serum-free medium containing no biotin, 40 µM biotin, or 40 µM biotin together with 100 µM of furin inhibitor I (Sigma) was added to the flask for 4 h. At the end of the incubation time, cells were harvested and lysed in RIPA buffer (Thermo Fisher Scientific) supplemented with protease and phosphatase inhibitors (Roche, Basel, Switzerland). Additionally, cell culture supernatants were collected and concentrated ~100-fold in a Amicon 50 kDa filter unit (Sigma) according to the manufacturer’s instructions. Cell lysate and concentrated supernatant protein concentrations were determined using a BCA^TM^ protein assay kit (Thermo Fisher Scientific). Next, 10 µg of total protein content from lysates and supernatants was resolved on NuPAGE^TM^ 4–12% Bis-Tris gels (Invitrogen, Carlsbad, CA, USA) under reducing conditions and transferred to a nitrocellulose membrane (BioRad, Hercules, CA, USA) using a BioRad system. The membranes were blocked for 1 h with 0.01% Tween-20/5% non-fat dry milk/TBS, and the primary antibodies were added overnight under agitation at 4 °C. The membranes were rinsed with 0.01% Tween-20/5% non-fat dry milk/TBS and incubated for 1 h with secondary antibodies under agitation at 4 °C. The membranes were then washed 3 times for 5 min with 0.01% Tween-20/TBS, and the peroxidase activity was evaluated with Amersham ECL Primer Western Blotting Detection Reagent (GE Healthcare, Little Chalfont, UK) on ImageQuant LAS4000 (GE Healthcare).

### 2.6. Flow Cytometric Validation of Antibody Reactivity

U2OS clone #7 cell culture supernatants were prepared as described above and used to assess the binding of the RUSH antibody to PD-L1-expressing cells through flow cytometry. Three million CHO-K1 and CHO PD-L1 cells were harvested, rinsed with PBS, and incubated for 30 min at 4 °C under agitation in the dark with either 150 µL of the recombinant Atezolizumab prepared in Sanofi research labs or concentrated U2OS clone #7 cell culture supernatant. Cells were then rinsed with PBS and secondarily stained for 30 min at 4 °C under agitation in the dark with an Alexa Fluor 647-coupled anti-human IgG antibody, before flow cytometry analysis of 100 µL solution on a MACSQuant (Miltenyi Biotec, Bergisch Gladbach, Germany).

### 2.7. High-Throughput Compound Screening for Modulators of Protein Secretion

U2OS clone #7 cells were seeded in black 384-well imaging plates (Greiner bio one), at 2000 cells/well, and incubated for 24 h at 37 °C in a humidified incubator with 5% CO_2_. Cells were treated on the next day with compounds from either the Prestwick library at 20 µM or 10 µM, the ICCB Known Bioactives library at 1/100^e^ or 1/1000^e^, or the autophagy compound library at 10 µM or 1 µM for 4 h. At the end of the incubation time, biotin was added at 40 µM for 1 h (untreated cells and DMSO-treated cells were used as a control). Cells were then fixed as described above and processed for subsequent automated image acquisition using a robot-assisted IXM XL BioImager (Molecular Devices, Sunnyvale, CA, USA) equipped with a Sola light source (Lumencor, Beaverton, OR, USA), adapted excitation and emission filters (Semrock, Rochester, NY, USA), and a 16-bit monochrome sCMOS PCO.edge 5.5 camera (PCO, Kelheim, Germany). A 20 X PlanAPO objective (Nikon, Tokyo, Japan) was used to acquire a minimum of 4 view fields in each well. The acquired images were processed using the open-access R software (https://www.r-project.org, accessed on 12 May 2023) with the use of the freely available packages EBImage (available on the Bioconductor repository https://www.bioconductor.org, accessed on 12 May 2023) and RBioFormats (https://github.com/aoles/RBioFormats, accessed on 12 May 2023), and the custom packages MetaxpR. (https://github.com/asauvat/MetaxpR, accessed on 12 May 2023) and MorphR (https://github.com/kroemerlab/MorphR, accessed on 12 May 2023). Nuclei were first detected using the Hoechst 33342 signal and used as a marker to detect cytoplasmic areas based on GFP signal, allowing for the evaluation of cytoplasmic GFP intensity. Data were subsequently extracted and statistically evaluated using R (https://www.r-project.org, accessed on 12 May 2023). Data were normalized using negative and positive controls, reduced, and transformed into a Z-score. The experiments were repeated manually at low scale with selected drugs, to confirm their effects on secretion; in this second set of assays, U2OS clone #7 cells incubated with secretion inhibitors were fixed and stained with Hoechst as described above, for imaging and viability determination based on cell counts.

### 2.8. ELISA

U2OS clone #7 cells were seeded in 6-well plates and kept for 24 h at 37 °C in a humidified incubator with 5% CO_2_. The next day, the supernatants were removed, cells were rinsed with 37 °C pre-heated PBS, and new medium containing the chemical treatment was added for 4 h. At the end of the incubation time, no biotin or 40 µM biotin was added to the flask for 30 min (inhibitory selected molecules) or 1 h (activator selected molecules). Then supernatants were collected and used for the ELISA assay, as follows: MaxiSorp 96-well plates (Thermo Fisher Scientific) were coated with 0.5 µg/mL hFc-human-PD-L1 (R&D Systems, Minneapolis, MN, USA) in PBS for 5.5 h at RT. Coating solution was then removed and 2% BSA/PBS blocking solution was added overnight at 4 °C. The next day, wells were washed 3 times with 0.5% BSA/0.05% Tween-20/PBS solution. Supernatants were added after a 1/3 dilution in 0.5% BSA/0.05% Tween-20/PBS, for 2.3 h at RT. Wells were washed 3 times with 0.5% BSA/0.05% Tween-20/PBS solution. Anti-human kappa light chain HRP-coupled antibody was added diluted in 0.5% BSA/0.05% Tween-20/PBS at 1/4000^e^ for 2.3 h at RT. Wells were washed 3 times with 0.5% BSA/0.05% Tween-20/PBS solution, before incubation with a substrate reagents pack (R&D Systems) for 15 min in the dark. Then, 50 µL of STOP solution (R&D Systems) was added to each well and the absorbance in each well was measured at 450 nm, and 635 nm for background subtraction, using a VICTOR^TM^ X4 (PerkinElmer, Waltham, MA, USA).

### 2.9. Statistics

Unless otherwise specified, data are reported as the mean ± SD of two replicates in a minimum of three independent experiments. Statistical significance was assessed using a one-sided Mann–Whitney U-test.

## 3. Results

### 3.1. Design of an Antibody RUSH System

**Figure 1:**
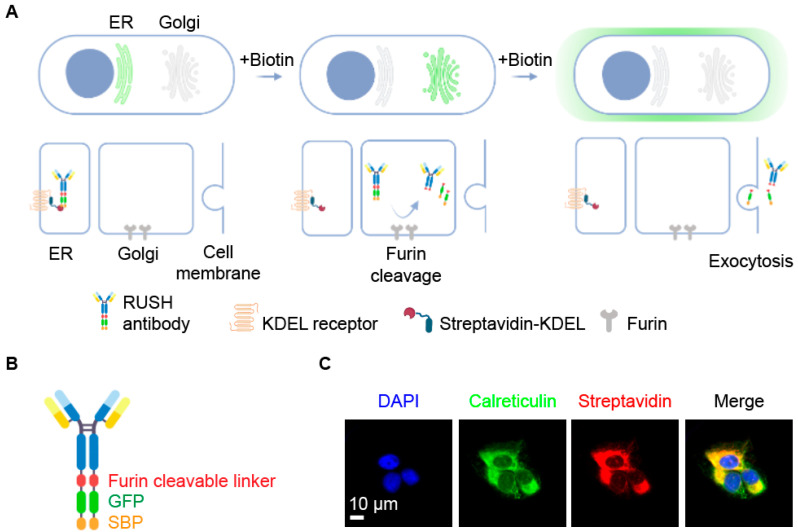
Design of the RUSH antibody secretion system (**A**) Principle of the RUSH assay for antibody secretion. The fusion protein (in this case, the antibody fused to the streptavidin binding domain, SBD, separated by a linker that contains a furin cleavage site) is retained in the ER lumen via the interaction of SBP with a streptavidin-KDEL hook. Upon addition of biotin, the interaction between the hook and the fusion protein is competitively disrupted, and the fusion protein is hence free to follow the classical secretory pathway to the Golgi, where the resident furin protease cleaves the linker between SBD and the antibody. The resulting parts are then secreted in the extracellular environment. The GFP moiety enables following the process with fluorescence microscopy. (**B**) Schematic representation of the custom anti-PD-L1 RUSH antibody, a fusion protein composed of an anti-PD-L1 IgG modified in its C-terminal end by the addition of a furin cleavable linker, a GFP, and an SBP sequence. (**C**) Immunostaining of fixed cells showing the colocalization of the streptavidin-KDEL hook with calreticulin in the ER. Pearson’s correlation coefficient (PCC) = 0.69. Surface overlap coefficient (SOC) = 0.45.

The RUSH system is composed of a hook that is streptavidin and a bait that is a protein fused to a streptavidin-binding peptide (SBP). When these two molecules are co-expressed, the bait is sequestered by the hook, due to the high-affinity streptavidin/SBP interaction (K_d_ in the range of 10^−8^ to 10^−9^ Mol) [27], and released upon addition of biotin, which outcompetes SBP due to its higher affinity for streptavidin (K_d_ in the range 10^−14^ Mol) [28]. Here, we adapted this cell-based assay to monitor the secretion kinetics of an antibody (the bait) and screen small compound libraries to identify secretion modulators. As antibodies are secreted through the conventional pathway, the streptavidin hook was fused to a KDEL peptide to ensure its retention in the endoplasmic reticulum (ER) through the interaction with the KDEL receptors present in the ER [29]. The antibody was also modified to fit the purpose of the assay. A polycistronic vector was used that encodes both the heavy and light (kappa) chains of a human PD-L1-specific antibody [30]. The C-terminus of the heavy chain was fused to a (Gly_4_Ser)_2_ linker, a furin-cleavable site, the green fluorescent protein (GFP), and SBP (Figure 1B). We reasoned that the fusion molecule (hereafter called RUSH antibody) would assemble into mature homodimers upon protein synthesis and remain in the ER due to its interaction with the streptavidin KDEL hook, until addition of biotin would trigger its release and maturation in the Golgi through its trafficking to the extracellular space (Figure 1A).

**Figure 2:**
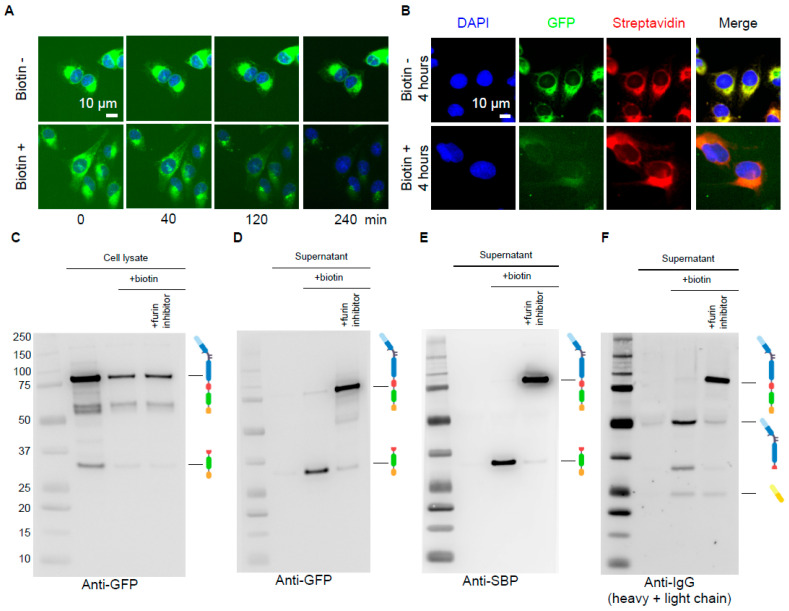
Characterization of the anti-PD-L1 antibody RUSH system (**A**) Time lapse microscopy of clone #7 cells incubated in the absence or in presence of 40 µM biotin, fixed and stained with DAPI. The secretion rate could be deducted from the decreasing intracellular GFP intensity upon addition of biotin to the cell cultures. (**B**) Immunostaining of fixed cells incubated for 4 h in the absence or in the presence of biotin showing the colocalization of the streptavidin-KDEL hook with the GFP-tagged antibody in the absence of biotin and the loss of colocalization 4 h post biotin addition. PCC = 0.51 in the absence of biotin, and PCC = 0.29 in the presence of biotin. (**C**–**F**) Western blot of cell lysates (**C**) or concentrated supernatants (**D**–**F**) of clone #7 incubated for 4 h in the absence of biotin, in the presence of 40 µM biotin, or in the presence of 40 µM biotin together with 100 µM furin inhibitor I. Membranes were probed with the indicated antibodies to detect proteins that contain the GFP moiety (**C**,**D**), the SBP moiety (**E**), or epitopes from the immunoglobulin heavy and light chains (**F**). Molecular mass standards (in kDa) are indicated on the left, in panel (**D**). The structures of the proteins detected by immunoblot are indicated based on the scheme shown in Figure 1B (blue, antibody heavy chain; yellow, antibody light chain; green, GFP; orange, SBP; red shape, linker with furin cleavage site; truncated red shape, linker residues left after cleavage by furin). Results are representative of at least three independent experiments.

The hook and bait expressing clone was obtained using U2OS cell transfection with the hook-encoding construct and the selection a clone with high hook expression levels, in order to ensure efficient retention of the bait and minimize leakiness in the absence of biotin. As shown in Figure 1C, Clone #BD6 staining with streptavidin-specific antibodies revealed the colocalization of the hook with the prototypic ER-sessile protein calreticulin [31]. Clone #BD6 was further transfected with the RUSH antibody encoding vector, followed by single cell cloning and the selection of the hook/bait clone used in this study (clone #7). This clone was selected based on two criteria: (i) a bright GFP-dependent fluorescent signal, indicating high abundance of the RUSH antibody; and (ii) discrete localization of the GFP signal in the perinuclear area, indicating ER retention (Figure 2A). Fluorescence video-microscopic observation of cells from clone #7 revealed that GFP remained in the same subcellular compartment over the 2 h of acquisition. On the contrary, the addition of biotin resulted in the relocation of the GFP signal towards discrete puncta that corresponded to the Golgi apparatus within 40 to 120 min, with a complete cytoplasmic GFP signal decay within 4 h, most likely due to the release of the fusion protein into the extracellular space (Figure 2A). The GFP signal co-localized with streptavidin at baseline and the addition of biotin did not modify this pattern, confirming the retention of the hook in the ER, regardless of the presence or absence of biotin in the medium. However, the GFP/streptavidin co-localization was lost 4 h after addition of biotin (Figure 2B). These results confirmed the expression of the bait and its retention in the ER through the hook, and suggest its progressive release in the extracellular space upon addition of biotin.

### 3.2. Biochemical Validation of the Antibody RUSH System

In the next step, we proceeded to the biochemical validation of clone #7, before its use in a screening campaign. For this aim, we characterized the molecular species synthesized and released by clone #7 cultured with or without biotin and a furin protease inhibitor. As can be seen in Figure 2C, the GFP-specific antibodies used in the Western blotting experiments revealed the presence of a major molecular species of ~80 kDa and two minor species of ~55 and ~35 kDa in the lysate of cells grown in the absence of biotin. The 80 kDa mass was compatible with the full-length heavy chain of the RUSH antibody (theoretical mass of 84 kDa), while the 35 kDa mass was compatible with the GFP-SBP C-terminal fragment of the heavy chain, which was released upon cleavage by furin (theoretical mass of 32 kDa). A similar analysis performed on the cell culture supernatant with GFP, SBP, and human IgG- specific antibodies only revealed trace amounts of recombinant protein in the absence of biotin (Figure 2D–F). This is consistent with the minor processing observed in cell lysates and therefore the efficient retention of the RUSH antibody in pre-Golgi intracellular compartments, as documented through the immunofluorescence (Figure 2A,B).

When cells were cultured in the presence of biotin, the Western blotting pattern in the cell lysate was unchanged, except that the intensity of the signal was lower, which was concomitant with the detection of recombinant species in the cell culture supernatant (Figure 2C–F). This demonstrates that the intracytoplasmic GFP decay observed via immunofluorescence in Figure 2A,B was a direct consequence of the protein release in the extracellular space. Moreover, the molecular masses of the material detected in the supernatant suggest an almost full processing of the released RUSH antibody. This was further confirmed by the comparable patterns obtained in cell lysates and in the cell culture supernatant when cells were grown in the presence of furin inhibitor. It can therefore be concluded that the RUSH antibody was released through the Golgi network and processed upon addition of biotin.

Taken together, these findings demonstrated that clone #7 allowed for biotin-stimulated release, as well as for the furin-dependent proteolytic maturation, of the RUSH antibody.

### 3.3. Immunological Validation of the Antibody RUSH System

**Figure 3:**
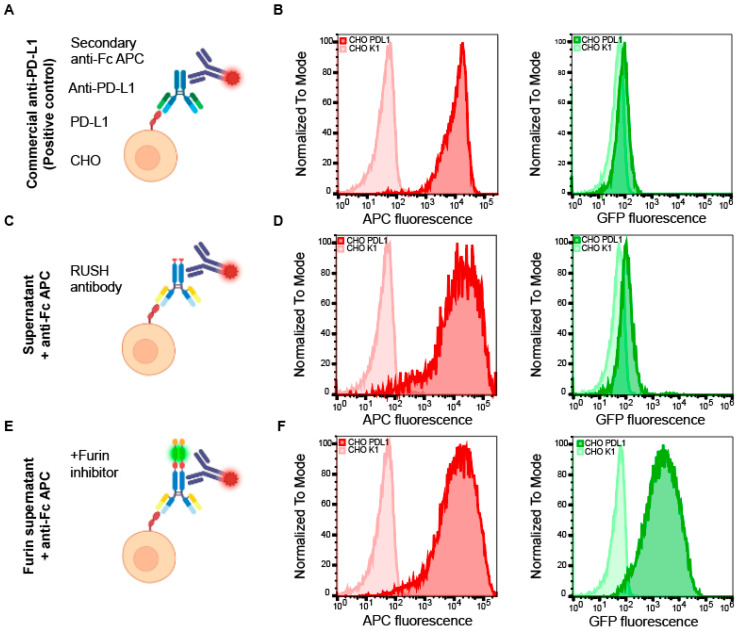
Characterization of the binding capacity of the anti-PD-L1 RUSH antibody released through the biotin-activated RUSH system. (**A**,**C**,**E**) Schematic representation of the antibody binding to PD-L1^pos^ cells and detection by flow cytometry. (**B**) FACS histogram plot of native recombinant and purified anti-PD-L1 antibody. (**D**,**F**) FACS histogram plots of processed and unprocessed RUSH anti-PD-L1 antibody released by clone #7 in the presence of biotin and in the absence or presence of furin inhibitor, respectively. Dark colors, binding to PD-L1^pos^ CHO cells (CHO-PDL1); light colors, binding to PD-L1^neg^ parental CHO cells (CHO-K1). Antibody binding was detected by means of an indirect immunofluorescence assay with a secondary anti-Fc APC conjugated (red) or by direct measurement of GFP fluorescence.

Besides the mechanistic validation of clone #7, in terms of the antibody production, retention, maturation, and release upon addition of biotin, we also addressed the PD-L1 binding properties of the secreted RUSH antibody. We further investigated the RUSH antibody binding properties to PD-L1 in its native conformation when it was presented at the plasma membrane. For this aim, clone #7 was incubated with biotin and the cell culture supernatant was assayed for binding on PD-L1^pos^ and PD-L1^neg^ CHO cells by FACS. As can be seen in Figure 3A,B, native recombinant anti-PD-L1 binding to PD-L1^pos^ (CHO-PDL1) but not PD-L1^neg^ CHO (CHO-K1) cells was documented, which validated the use of such a system for further characterization of the RUSH antibody. Similarly, the supernatant of clone #7 cultured in the presence of biotin demonstrated binding activity to CHO-PDL1 cells but not to CHO-K1 cells (Figure 3C,D), indicating that the released RUSH antibody conserved its antigen-binding properties, despite the presence of extra ammino-acids at its C-terminus, left after processing with furins. Of note, PD-L1 binding was also detectable in the supernatant of clone #7 cultured in the presence of biotin and a furin protease inhibitor (Figure 3E,F). In these conditions, binding was detected through both the secondary staining antibody and the GFP moiety of the unprocessed RUSH antibody, which was not detectable with the native antibody (Figure 3B) and only very slightly detected in the case of the supernatant of clone #7 cultured with biotin only (Figure 3D). Altogether, these observations indicate that clone #7 was fit for both screening campaigns and further antibody characterization in PD-L1-binding assays.

### 3.4. Pharmacological Screen for Modulators of Biotin-Induced Antibody Release

**Figure 4:**
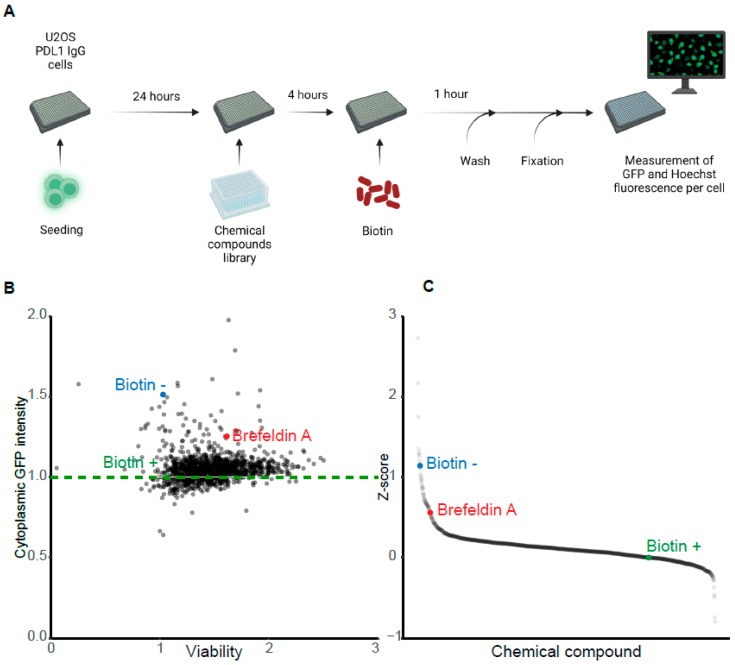
High-throughput screening of chemical compound libraries for the identification of modulators of protein secretion. (**A**) Screening workflow. U2OS clone #7 was plated in 384-well plates and incubated for 24 h, before treatment with each compound of the Prestwick, ICCB Known Bioactives, or autophagy compounds at two concentrations: low and high (with a 10-fold difference). Biotin was added after 4 h, and cells were washed and fixed 1 h after addition of biotin and processed for automated image acquisition of the intracellular Hoechst 33324 and GFP fluorescence. (**B**) Dot plot representation of drugs based on their impact on GFP release (cytoplasmic GFP signal) and nucleus integrity (viability). (**C**) Z-score ranking of the cytoplasmic GFP intensity after treatment. The results of the Prestwick library screening at a low drug concentration were plotted. Brefeldin A was used as a positive control for the inhibition of secretion. The compounds causing a cytoplasmic GFP intensity below that observed for the biotin-only treated cells were potential enhancers of protein secretion. Drugs that induced a GFP fluorescence level higher than that measured in the presence of biotin only were potential inhibitors of protein secretion.

We next sought to use clone #7 in screening campaigns for the identification of drugs capable of enhancing or inhibiting antibody secretion. For this, clone #7 was cultured in 384 well plates and treated for 4 h with drugs from chemical libraries, followed by the addition of biotin for 1 h and the determination of the intra-cytoplasmic GFP signal (Figure 4A). Cell counting was facilitated by nuclear counterstaining with Hoechst 33324, in order to assess the viability and distinguish drug effects on the secretion kinetics from direct cytotoxicity, and to select relevant drugs for further characterization. Two independent screening campaigns were performed, to test a total of 2300 drugs at 2 different concentrations (i.e., a low concentration and a 10-fold higher concentration, referred to as “low” and “high” doses, as described in the Section 2).

**Figure 5:**
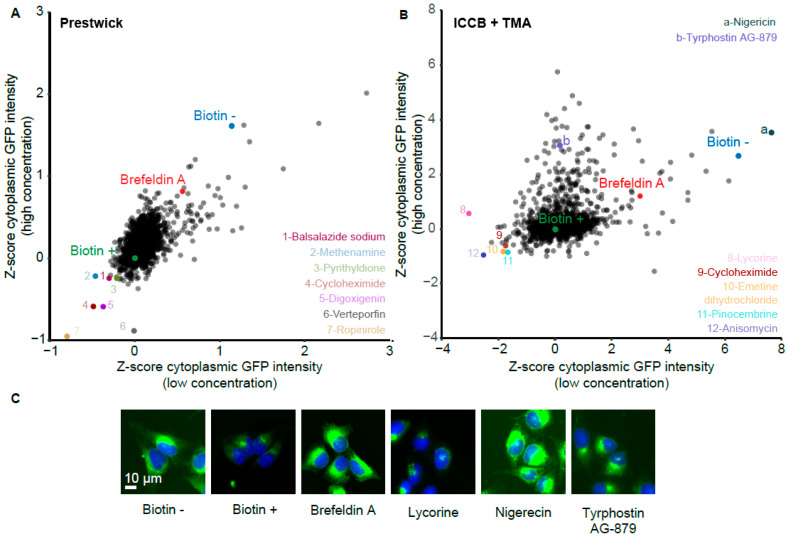
Selection of potential modulators of protein secretion. (**A**,**B**) Dot plot representation of drug efficiency based on the Z-scores measured at low and high drug concentration ((**A**) Prestwick library; (**B**) ICCB Known Bioactives and TargetMol Autophagy libraries). Compounds selected for further characterization are listed and highlighted in color. (**C**) Clone #7 was treated with the indicated compounds, fixed and analyzed as described in Figure 4A legend.

The biotin-induced decrease of the cellular GFP signal was influenced by a minority of compounds from various chemical libraries, as indicated through the plotting of primary data (Figure 4B) or Z-score analyses (Figure 4C). As an internal control, brefeldin A, which is an inhibitor of conventional Golgi-dependent protein secretion [32], inhibited the biotin-induced decrease of the GFP signal (Figure 4B,C). Plotting of the Z-scores obtained at the low and high concentrations for each compound (Figure 5A,B) led to the identification of several compounds that consistently decreased the fluorescent signal, therefore acting as secretion activators. In order to select candidates to be further characterized, the 20 best activators identified at either the low or high concentration were identified, and among these, activators working at both concentrations were shortlisted. In the Prestwick library, balsalazide sodium (no longer commercially available), methenamine, pyrithyldione, cycloheximide, digoxigenin, verteporfin, and ropinirole fulfilled these criteria (Figure 5A). Verteporfin was eliminated due to its phototoxicity that leads to GFP bleaching [33]. In a combined screen involving compounds contained in two additional libraries (ICCB Known Bioactives and TargetMol Autophagy), several additional compounds (anisomycin, emetine, and pinocembrine) were active at both low and high concentrations. Additionally, although lycorine was not among the 20 most active compounds at high concentration, it was more efficient than any other compound at low concentration and was therefore selected (Figure 5B).

Several drugs were also identified that inhibited the biotin-induced cytoplasmic GFP signal decay, therefore acting as potential secretion inhibitors, among which nigericin and tyrphostin AG-879 were selected for further characterization (Figure 5B) because they had not been identified previously by Zhao et al. [26].

### 3.5. Validation of Stimulators and Inhibitors of Biotin-Induced Antibody Release

**Figure 6:**
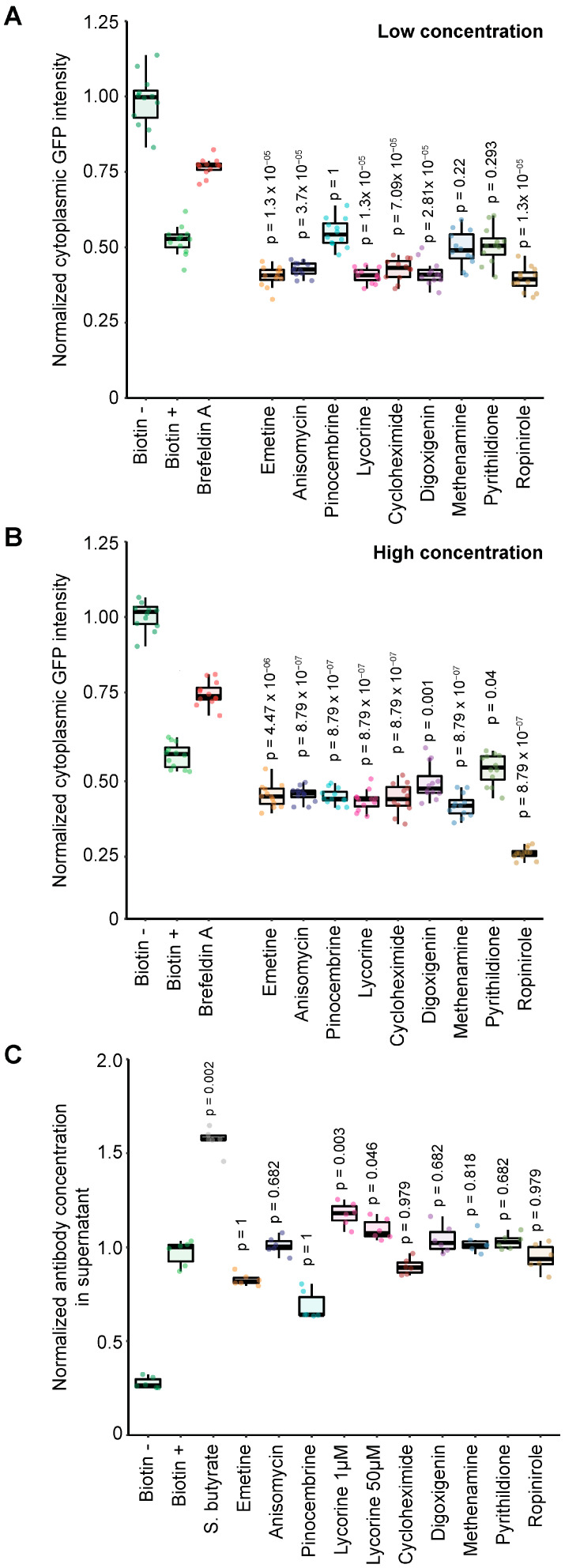
Validation of enhancers of protein secretion. (**A**,**B**) Clone #7 cells were treated with the indicated compounds and analyzed as indicated in the legend of Figure 4A. Drugs were used at low (**A**) or high concentration (**B**), as indicated in the text, and the post-treatment intracytoplasmic fluorescence was normalized to that measured in mock-treated cells (no biotin). (**C**) ELISA assay on immobilized PD-L1. Clone #7 cells were treated with the indicated compounds, and supernatants were assayed for PD-L1 binding. Sodium butyrate was used as a protein secretion enhancer positive control.

Medium- and high-throughput screenings often yield false positive and negative hits, calling for independent validation in low-throughput experiments with orthogonal technologies. In a first round of experiments, we evaluated the effects of shortlisted compounds with potential antibody secretion enhancer activity. The compounds identified in the screening campaigns were retested on clone #7 at a low concentration (Figure 6A) or a ten-fold higher concentration (Figure 6B) for their ability to increase antibody secretion. This experiment showed that all selected hits were active at the highest concentration, which confirmed the results of the screening (Figure 6B). We further characterized these hits in an PD-L1 binding ELISA. In this system, sodium butyrate induced a two-fold increase of RUSH antibody release upon biotin addition, as expected from its effect on protein expression [34,35]. Among the drugs tested, only lycorine (tested at both 1 and 50 µM) was considered to be active, although its effect was milder than that of sodium butyrate, while all the other compounds failed to display any antibody secretion stimulatory activity (Figure 6B).

**Figure 7:**
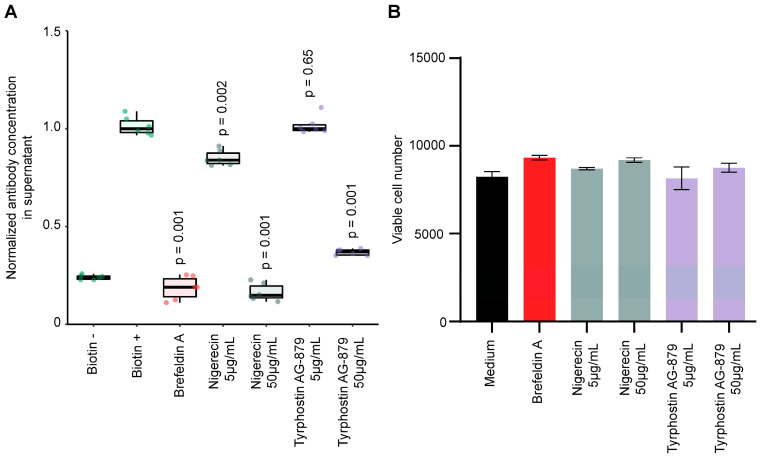
Validation of inhibitors of protein secretion. (**A**) ELISA assay on immobilized PD-L1. Clone #7 cells were treated with the indicated compounds and supernatants were assayed for PD-L1 binding. Brefeldin A was used as a protein secretion inhibitor positive control. (**B**) Cells treated in A were subjected to a toxicity assay. Hoechst 33324 fluorescence was measured, which enabled counting the adherent living cells in each well.

Similarly, potential secretion inhibitors identified during the screening campaigns were also tested in an ad hoc assay. Both nigericin and tyrphostin AG-879 (used at 50 µg/mL) turned out to be as potent as brefeldin A in suppressing antibody secretion (Figure 7A), without affecting cellular viability (Figure 7B). Cell imaging further confirmed the secretion activator effect of lycorine and the inhibitory effect of nigericin and tyrphostin AG-879 (Figure 5C).

## 4. Discussion

In the present study, we provide evidence that the RUSH system can be adapted to the controlled release of functional antibodies (exemplified by the immunotherapeutic anti-PD-L1 antibody Atezolizumab) from cultured U2OS cells via the addition of biotin. Using this antibody compatible RUSH system, small chemical compounds libraries were successfully screened using a high-throughput fluorescence imaging system, for the identification of modulators of conventional antibody secretion. Here, we report one novel activator and two novel inhibitors of antibody secretion validated using enzyme-linked immunosorbent assay (ELISA) antibody titration.

The RUSH system offers a cost-effective, high-throughput, high-content, and screening-compatible alternative to complementary assays such as ELISA. Thus, expressed in firmly adherent human osteosarcoma U2OS cells, the RUSH system becomes a valuable tool for the image-based kinetic assessment of protein trafficking. Of note, U2OS cells are neither antibody producing nor specialized secretory cells, which necessitates validation in physiologically relevant systems. In previous publications, variations of the RUSH system were generated by fusing different proteins, such as calreticulin [36], giantin [37], transferrin receptor [38], and tumor necrosis factor [37] to SBP and a reporter protein, either GFP, which can be directly visualized by fluorescence microscopy, or other non-fluorescent proteins tracked by alternative methods. The particularity of these model cargos is their monomeric nature, and no large homo-dimeric proteins such as antibodies have ever been studied in the RUSH system, in order to document whether secretion can be triggered and whether antibodies maintain antigen-binding properties upon release after long-term immobilization in the ER lumen. These questions needed to be addressed prior to using the RUSH system for the identification of antibody secretion modulators in screening campaigns.

Setting up a RUSH system requires the addition of SBP to the antibody for reversible ER targeting and a fluorescent protein (e.g., GFP) for automatized video microscopy monitoring of antibody secretion. As highlighted by Haas et al. and Luria et al., this represents a challenge for antibodies, since immunoglobulins and GFP do not normally originate from the same cellular compartment, and the addition of foreign sequences to antibodies has been shown to interfere with their secretion and functionality [39,40]. The mechanism of IgG heavy and light chain assembly prior to mature antibody secretion poses another challenge. Indeed, while antibody heavy chains require assembly with light chains for secretion, a free light chain might be secreted. We therefore opted to retain the RUSH antibody through its heavy chain, in order to confidently monitor the secretion of fully assembled antibodies [41,42,43,44]. With these constraints considered, the RUSH machinery was added to the C-terminus of the anti-PD-L1 heavy chain. A furin cleavage site was also added between the heavy chain and the RUSH construct for the removal of the SBP and GFP sequences past the Golgi network, which might have affected the functions of the antibody otherwise.

Our data showed the efficient detection and retention of the antibody-GFP fusion in the ER of clone #7, and its release and maturation upon addition of biotin in the cell culture medium. ELISA and cell-based assays also demonstrated the specific binding of the released antibody to its PD-L1 target. The fact that the majority of secreted molecules were processed by furins suggests that the secretory machinery of clone #7 was not saturated, which makes this system relevant for the identification of secretion modulators. Of note, the uncleaved full-length antibody retained its specificity and was detectable through its GFP moiety. This has also been described by others and confirms the potential interest of such constructs for live imaging of cells, organs, or organisms [40].

Using this antibody-compatible RUSH system, we screened chemical libraries to identify small molecules that would modulate antibody secretion. The conditions were adapted to identify inhibitors of protein secretion and compare the results with previous screening campaigns performed with a distinct cargo [26], but also with activators. Among the 31 inhibitory compounds of the Prestwick library identified by Zhao et al., 13 were also identified in our screen, due to their ability to increase intracytoplasmic GFP intensity either at high or low concentration, suggesting consistency between the two approaches, despite the use of different clones and cargos. Among the two unique compounds that caused the retention of the RUSH antibody in the cytoplasm, tyrphostin AG-879, which is characterized as a tyrosine kinase inhibitor (IC_50_ = 10 µM), could be validated as an inhibitor of antibody secretion when it was used at a concentration of 50 µg/mL. The identification of a kinase inhibitor is consistent with the involvement of various kinases that act on the Golgi and that are responsible for protein maturation, secretion, and triage to the degradation pathways [45]. This is also consistent with the need for phosphorylation of effectors of the secretory pathway, such as the eukaryotic initiation factor 2 (eiF2), resulting in the decrease of the UPR and hence of the secretory capacity of the cells [46]. Nigericin was also confirmed as a secretion inhibitor when used at a concentration of 50 µg/mL (but not 5 µg/mL). This potassium ionophore might interfere with bioenergetic functions, pH regulation, and ion homeostasis, and induce pleiotropic cellular effects, leading to a decreased secretion potential [47].

Our screening campaigns also led to the identification of candidate molecules that might increase antibody secretion in the biotin-stimulated RUSH system, because they decreased the intracytoplasmic GFP fluorescence signal, among which eight compounds (anisomycin, cycloheximide, digoxigenin, lycorine, methenamine, ropinirole, pinocembrine, and pyrithyldione) were selected for further characterization by ELISA. This assay measures the RUSH antibody binding to its immobilized PD-L1 target and was used to measure antibody titers in cells treated with candidate molecules. Unexpectedly, among these eight compounds that consistently decreased intracytoplasmic GFP fluorescence, which was interpreted as accelerated antibody release, only lycorine was shown to increase RUSH antibody titers in treated cell culture supernatant. These discrepancies might be attributable to the nature of the read out of both assays, with one being the measurement of intracytoplasmic GFP fluorescence intensity decrease and the second being the antibody titration in the cell culture supernatant. As a possible explanation, compounds selected based on the screening results might have had an unsuspected negative impact on the affinity of the RUSH antibody/antigen interaction, therefore masking their direct impact on antibody secretion. On the contrary, the fact that lycorine was validated in both assays and in several independent experiments strongly supports its stimulatory effect on antibody secretion in our system.

Lycorine likely has multiple effects on cell biology, with several cellular targets and a proposed utility for rather diverse indications, including cancer [48], fungal infection [49], and Alzheimer’s disease [50]; however, no effect on secretion has been published to the best of our knowledge. Further investigations will be required to decipher its mode of action as an activator, as well as that of the inhibitors identified in this work.

In future applications, the screening approach presented in this study could be upscaled to larger pharmacological screening campaigns at the industrial level, for the discovery of novel molecules with secretion modulatory effects. Potential clinical applications would involve the direct use of the identified compounds to modulate protein secretion in related diseases, or the identification of their cellular target for subsequent development of therapeutic solutions [5]. In an industrial context, the RUSH system adapted to producer cell lines in liquid culture could be seen as a tool to control the secretion of recombinant therapeutics proteins of interest with toxic properties once secreted in the culture supernatant, resulting in cell growth inhibition or even cell death. This would represent a valuable alternative to the inducible transcription systems that were initially explored for the decoupling of cell growth and product formation, which proved challenging to develop [51].

## 5. Conclusions

Overall, while the addition of lycorine in bioreactors might hamper drug substance purification in industrial processes, deciphering its mechanism of action would be of great interest. A direct action of lycorine on cellular targets through genetic engineering could unlock the secretory potential of cell hosts. Such hosts would bring added value to the field of recombinant therapeutic protein production and to the pharmaceutical industry.

## Data Availability

The data presented in this study are available on request from the corresponding authors. The data are not publicly available since they are being used for filing a patent.
